# Supplementary material for: Powassan Virus NS5 Antagonizes TYK2-Mediated Immune Signaling Pathways
Source: bioRxiv. 2026 Sep 8:2026.09.07.749923. Preprint. [Version 1] doi: 10.64898/2026.09.07.749923 (PMC13622581; doi:10.64898/2026.09.07.749923)
Supplement: Supplementary file 1 [file NIHPP2026.09.07.749923V1-supplement-1.pdf]

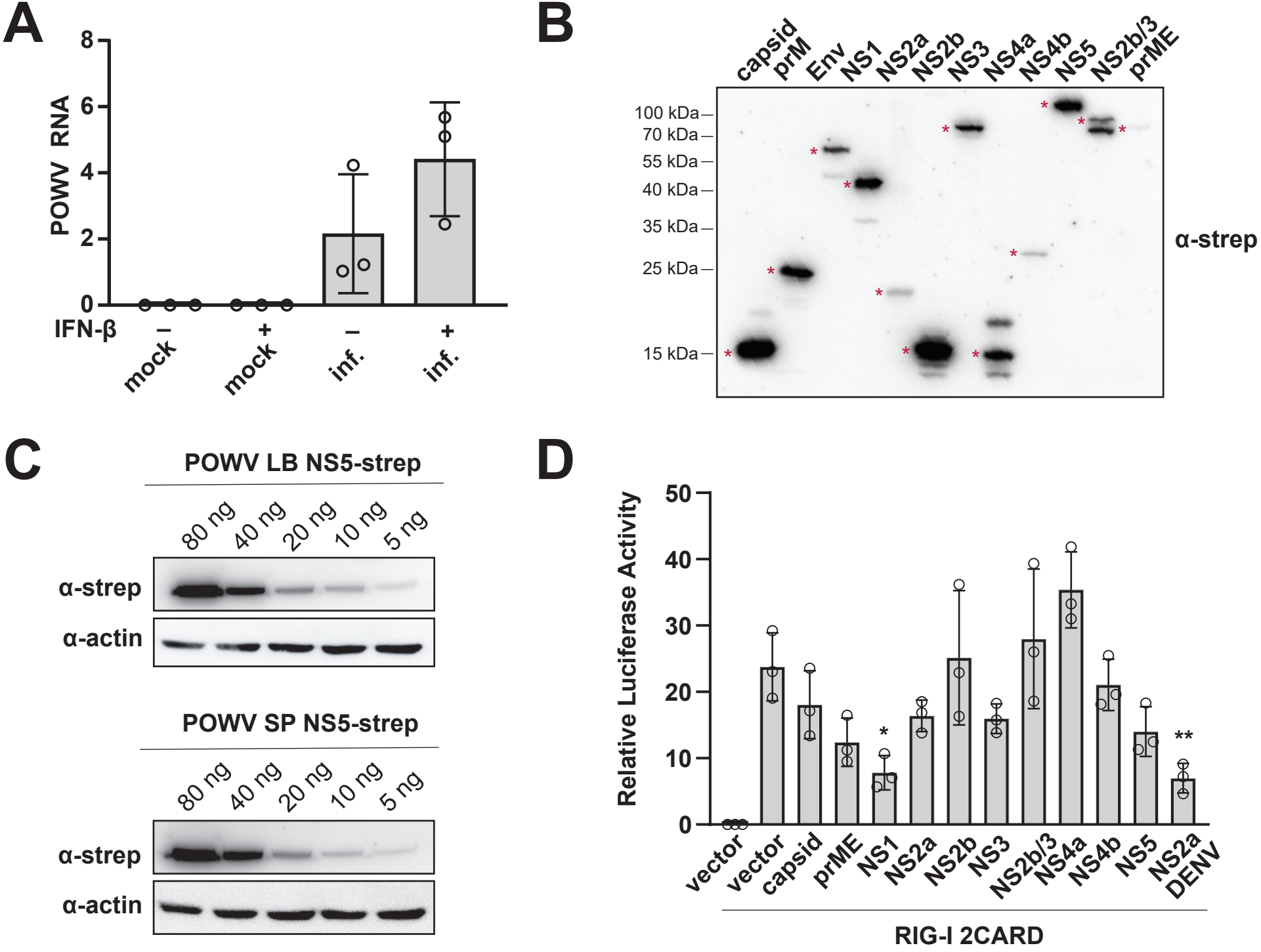

A
POWV RNA
IFN-β
mock
mock
inf.
inf.
B
capsid
prM
Env
NS1
NS2a
NS2b
NS3
NS4a
NS4b
NS5
NS2b/3
prME
100 kDa
70 kDa
55 kDa
40 kDa
35 kDa
25 kDa
15 kDa
α-strep
C
POWV LB NS5-strep
80 ng
40 ng
20 ng
10 ng
5 ng
α-strep
α-actin
POWV SP NS5-strep
α-strep
α-actin
D
Relative Luciferase Activity
vector
vector
capsid
prME
NS1
NS2a
NS2b
NS3
NS2b/3
NS4a
NS4b
NS5
NS2a DENV
RIG-I 2CARD

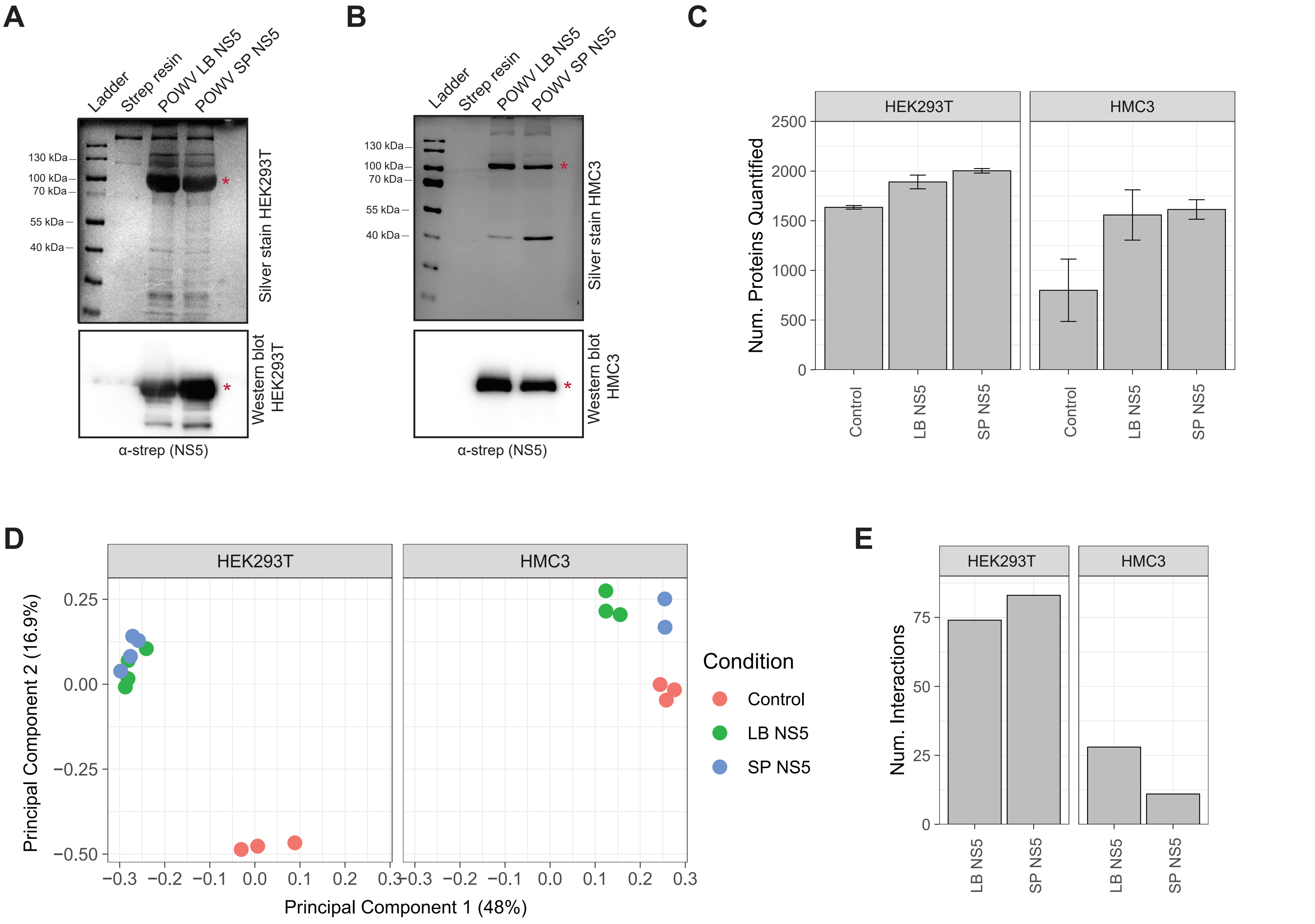

A
Ladder
Strep resin
POWV LB NS5
POWV SP NS5
130 kDa
100 kDa
70 kDa
55 kDa
40 kDa
*
Silver stain HEK293T
Western blot HEK293T
α-strep (NS5)
B
Ladder
Strep resin
POWV LB NS5
POWV SP NS5
130 kDa
100 kDa
70 kDa
55 kDa
40 kDa
Silver stain HMC3
Western blot HMC3
α-strep (NS5)
C
HEK293T
HMC3
Num. Proteins Quantified
2500
2000
1500
1000
500
0
Control
LB NS5
SP NS5
D
HEK293T
HMC3
Principal Component 2 (16.9%)
0.25
0.00
−0.25
−0.50
−0.3
−0.2
−0.1
0.0
0.1
0.2
0.3
Principal Component 1 (48%)
Condition
Control
LB NS5
SP NS5
E
HEK293T
HMC3
Num. Interactions
75
50
25
0
LB NS5
SP NS5

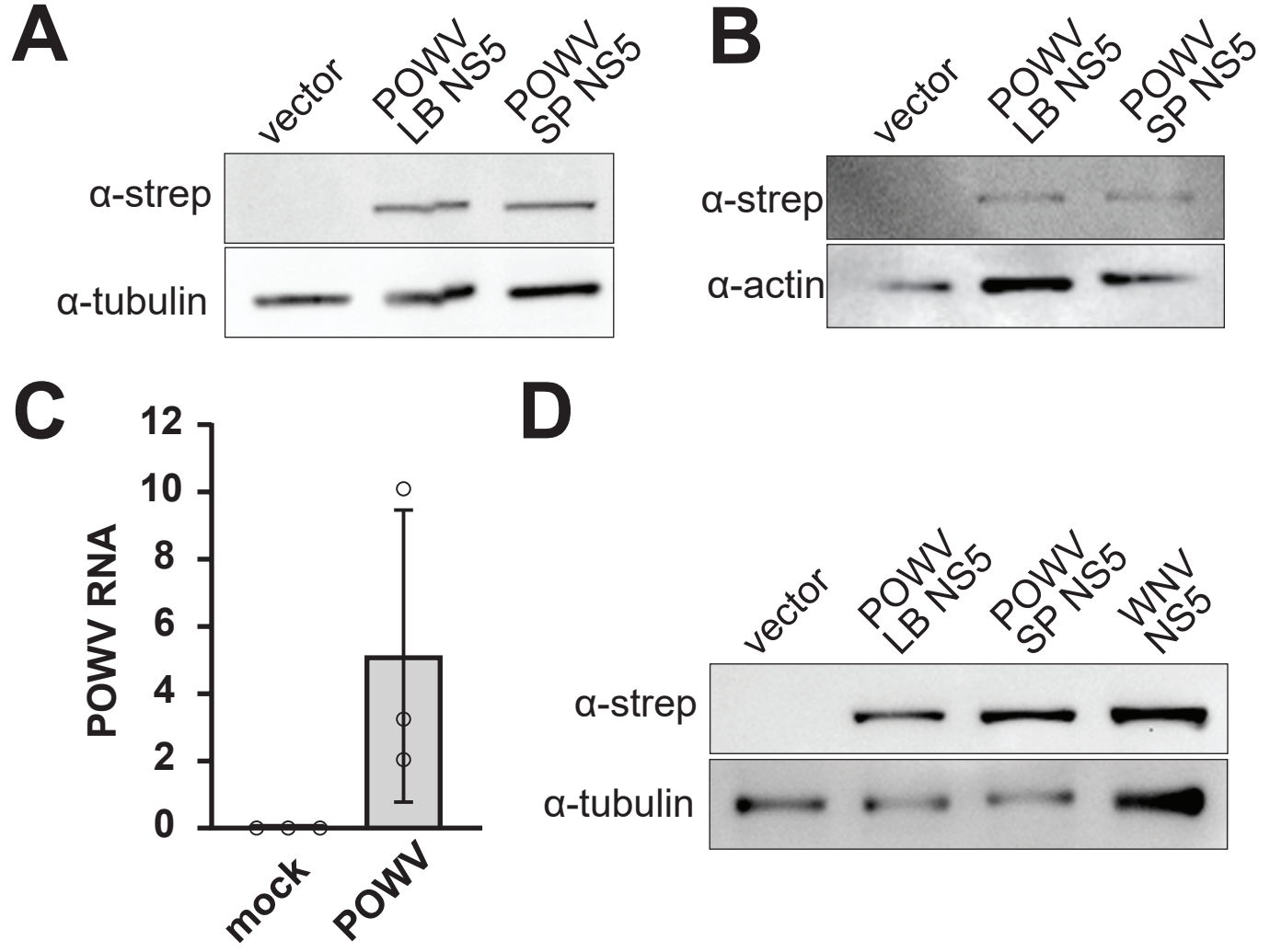
A
vector
POWV LB NS5
POWV SP NS5
α-strep
α-tubulin
B
vector
POWV LB NS5
POWV SP NS5
α-strep
α-actin
C
POWV RNA
12
10
8
6
4
2
0
mock
POWV
D
vector
POWV LB NS5
POWV SP NS5
WNV NS5
α-strep
α-tubulin
