# Supplementary material for: Powassan Virus NS5 Antagonizes TYK2-Mediated Immune Signaling Pathways
Source: bioRxiv. 2026 Sep 8:2026.09.07.749923. Preprint. [Version 1] doi: 10.64898/2026.09.07.749923 (PMC13622581; doi:10.64898/2026.09.07.749923)
Supplement: Supplementary file 2 [file media-3.pdf]

Table S2: Cloning and mutagenesis primers and gene blocks

**Primers used to clone POWV genes:**	
POWV-LB-Capsid-F	GAGAAGGGGGCGGCCATGGTGACTACTTCTAAAGGAAAGG
POWV-LB-Capsid-R	AAACGGGCCCTCTAGCTAAGCCATCAAAGCCATTACC
POWV-LB-PrM-Env-F	CAGTGTGGTGGAATTATGGCCATGGCGACCTCC
POWV-LB-PrM-Env-R	CTCCCTCGAGCGGCCCGGCGTATACAGGCCCAAGG
POWV-LB-NS1-F	CAGTGTGGTGGAATTATGGACTATGGATGCGCAGTTG
POWV-LB-NS1-R	CTCCCTCGAGCGGCCCAGCCATTACCATAGATCTAACCAG
POWV-LB-NS2a-F	CAGTGTGGTGGAATTATGGACAACGGCGCGATGC
POWV-LB-NS2a-R	CTCCCTCGAGCGGCCCTCGTCTGCCATGGCCCC
POWV-LB-NS2b-F	CAGTGTGGTGGAATTATGAGTCTGAGTGAACCCC
POWV-LB-NS2b-R	CTCCCTCGAGCGGCCCTCTGCGTGTTGACGAGAG
POWV-LB-NS3-F	TACCGAGCTCGGATCATGACTGACCTTGTATTCTCAGGGC
POWV-LB-NS3-R	CTCCCTCGAGCGGCCCCCTGCGGCCAGAAGCGTA
POWV-LB-NS4a-F	CAGTGTGGTGGAATTATGAGTGCCATGGACATCTTCACTG
POWV-LB-NS4a-R	CTCCCTCGAGCGGCCCGCGTTGCTTTCCCGGTTC
POWV-LBNS4b-F	CAGTGTGGTGGAATTATGAATGAACTGGGCTATTTGGAGC
POWV-LB-NS4b-R	CTCCCTCGAGCGGCCCCCTTCTAGCCCCCTGAGTTC
POWV-LB-NS5-F	TTAAACTTAAGCTTGATGGGTGGAGCAGAGGGAAG
POWV-LB-NS5-R	CTCCCTCGAGCGGCCCGATTATTGAGCTCTCTAGCTTGAG
POWV-SP-NS5-F	TTAAACTTAAGCTTGATGGGTGGAGCAGAGGGAAG
POWV-SP-NS5-R	CTCCCTCGAGCGGCCCGATTATTGAGCTCTCTAGCTTGAG

Primers used to clone genes into pCDH lentiviral expression plasmid:	
Lenti-EV-Fwd	GCGATATCGATCCGGCGCGCCCCTGCAGGTTAATTAAG
Lenti-EV-Rev	CCGGATCGATATCGCTAGCTCTAGAATCTTCCCTCCATA
Lenti-WNV NS5-Fwd	CATAGAAGATTCTAGAATGGGTGGGGCAAAAGGAC
Lenti-WNV NS5-Rev	AGATCCTTCGCGGCCGCTTAAACGGGCCCCTTCTCG
Lenti-POWV-LB NS5-Fwd	CATAGAAGATTCTAGAATGGGTGGAGCAGAGGGAAG
Lenti-POWV-LB NS5-Rev	AGATCCTTCGCGGCCGCTTAAACGGGCCCCTTCTCG
Lenti-POWV-SP NS5-Fwd	CATAGAAGATTCTAGAATGGGTGGAGCAGAGGGAAG
Lenti-POWV-SP NS5-Rev	AGATCCTTCGCGGCCGCTTAAACGGGCCCCTTCTCG
**Primers used to generate POWV-LB NS5 mutants:**	
POWV-LB NS5-Mtase-Fwd	GTTTAAACTTAAGCTATGGGTGGAGCAGAGGGA
POWV-LB NS5-Mtase-Rev	TCCCTCGAGCGGCCCGGATGCCGCTGATCCGG
POWV-LB NS5-RdRp-Fwd	GTTTAAACTTAAGCTATGCTGATCAACGGAGTGG
POWV-LB NS5-RdRp-Rev	CTCCCTCGAGCGGCCCGATTATCGAGCTCTCTAGCTTGAGCTC
POWV-LB NS5-RdRp-453-Rev	CTCCCTCGAGCGGCCCCCACTTATTCTGTTCATCCGACCAT
POWV-LB NS5-RdRp-553-Rev	CTCCCTCGAGCGGCCCTAGCAGCCAGCCCAGATAGTT
POWV-LB NS5-RdRp-603-Rev	CTCCCTCGAGCGGCCCTTGGACTTTCATGTTTGTGATGGTGTTGAGGG
POWV-LB NS5-RdRp-703-Rev	CTCCCTCGAGCGGCCCCTTCATCACAAGTTCATGGAAGTGATGTG
POWV-LB NS5-RdRp-803-Rev	CTCCCTCGAGCGGCCCCAAAATCCAGACTTTGTTCCAGATGCG
POWV-LB NS5-5A-Fwd	CTGCTCTGGCAGCCGTAGCAACCTGGTTGAAGGAGCACG
POWV-LB NS5-5A-Rev	CGGCTGCCAGAGCAGGGGCCTGTGAGTCAGCTGGTCC

POWV-LB NS5-WNV NS5 swap-Fwd	CTGCTCTGGCAGCCGTAGCAACCTGGTTGAAGGAGCACG
POWV-LB NS5-WNV NS5 swap-Rev	CGGCTGCCAGAGCAGGGGCCTGTGAGTCAGCTGGTCC
POWV-LB-WNV chimera gBlock Amp-Fwd	CAACTCATCCGCATGATGGAAGGAGAAGGAGTCATAGGAC
POWV-LB-WNV chimera gBlock Amp-Rev	ACAATCATCTCCACTCACAAGCATCCTCCCTAGTC

Table S3: Plasmids used in this study

Construct name	Source
pcDNA4_TO-Strep-empty vector	This study
pcDNA4_TO-Strep-GFP	
pcDNA4_TO-Strep-WNV-NS5	
pcDNA4_TO-POWV-Strep-Capsid	
pcDNA4_TO-POWV-LB PrM-Env-Strep	
pcDNA4_TO-POWV-LB NS1-Strep	
pcDNA4_TO-POWV-LB NS2a-Strep	
pcDNA4_TO-POWV-LB NS2b-3-Strep	
pcDNA4_TO-POWV-LB NS2b-Strep	
pcDNA4_TO-POWV-LB NS3-Strep	
pcDNA4_TO-POWV-LB NS4a-Strep	
pcDNA4_TO-POWV-LB NS4b-Strep	
pcDNA4_TO-POWV-LB NS5-Strep	
pcDNA4_TO-POWV-SP NS5-Strep	
pcDNA4_TO-POWV-LB NS5-Strep-MTase only	
pcDNA4_TO-POWV-LB NS5-Strep-RdRp only	
pcDNA4_TO-POWV-LB NS5-Strep-RdRp-453	
pcDNA4_TO-POWV-LB NS5-Strep-RdRp-553	
pcDNA4_TO-POWV-LB NS5-Strep-RdRp-603	
pcDNA4_TO-POWV-LB NS5-Strep-RdRp-703	
pcDNA4_TO-POWV-LB NS5-Strep-RdRp-803	

pcDNA4_TO-POWV-LB NS5-Strep-5A	
pcDNA4_TO-POWV-LB NS5-Strep-WNV swap	
pDEST40-TYK2-DDAA-V5	
pDEST40-TYK2-DDRR-V5	
pDEST40-TYK2-V5	Gracias et al., 2023
pDEST40-TYK2-N-V5	
pDEST40-TYK2-C-V5	
pDEST40-TYK2-delta-KL-V5	
pDEST40-TYK2-TK-V5	
pDEST40-TYK2-delta-TK-V5	
pDEST40-TYK2-KL-V5	
pMD2.G	Addgene #12259
psPAX2	Addgene #12260
pCDH_Str-Ii	Addgene #65313
pGL4.45[luc2P/ISRE/Hygro]	Promega #E4141
pNL1.1.TK[Nluc/TK]	Promega # N1501
RIG-I 2Card	Gift from Dr. Michaela Gack (PMID: 26998762)
IFN-β luciferase reporter	Addgene #102597
pCDH-empty vector-Strep	This study
pCDH-POWV-LB NS5-Strep	
pCDH-POWV-SP NS5-Strep	
pCDH-WNV NS5-Strep	

Table S4: Antibodies used in this study

Antibody	Source
Rabbit anti-TYK2	Cell Signaling Technology #9312S
Rabbit anti-pY-TYK2	Cell Signaling Technology #9321S
Mouse anti-IFNAR	Santa Cruz Biotechnology #sc-7391
Rabbit anti-STAT1	Cell Signaling Technology #14994S
Rabbit anti-pY-STAT1	Cell Signaling Technology #7649L
Rabbit anti-STAT2	Cell Signaling Technology #72604S
Rabbit anti-pY-STAT2	Cell Signaling Technology #88410S
Mouse anti-Strep tag	Abcam #184224
Rabbit anti-β-actin	CST #4967S
Mouse anti-α-tubulin	Sigma-Aldrich #T6199
Mouse anti-LGTV E (cross-reacts with POWV E)	BEI Resources #NR-40316
Rabbit anti-V5 tag	Bethyl # A190-120A
Mouse anti-V5 tag	Invitrogen #R960-25
HRP goat anti-Rabbit IgG (H+L)	Invitrogen #G-21234
HRP goat anti-Mouse IgG (H+L)	ThermoScientific #G-21040

Table S5: qRT-PCR primers used in this study

Target	Forward primer	Reverse primer
18S rRNA	GGCCCTGTAATTGGAATGAGTC	CCAAGATCCAACTACGAGCTT
POWV-SP	GCAGCACCATAGGTAGAATGT	CCACCCACTGAACCAAAGT
IFIT1	GCGCTGGGTATGCGATCTC	CAGCCTGCCTTAGGGGAA
MX1	GTTTCCGAAGTGGACATCGCA	CTGCACAGGTTGTTCTCAGC
RIG-I	CTGGACCCTACCTACATCCTG	GGCATCCAAAAAGCCACGG
SOCS1	CACGCACTTCCGCACATTC	TAAGGGCGAAAAAGCAGTTCC
